# Relationship between estrogen receptor α location and gene induction reveals the importance of downstream sites and cofactors

**DOI:** 10.1186/1471-2164-10-381

**Published:** 2009-08-18

**Authors:** Fabio Parisi, Bernhard Sonderegger, Pratyaksha Wirapati, Mauro Delorenzi, Felix Naef

**Affiliations:** 1 School of Life Sciences, Swiss Institute for Experimental Cancer Research (ISREC), Ecole Polytechnique Fédérale de Lausanne (EPFL), CH-1015 Lausanne, Switzerland; 2 Swiss Institute of Bioinformatics, CH-1015 Lausanne, Switzerland; 3 National Centers for Competence in Research (NCCR), Molecular Oncology, Ch. des Boveresses 155, 1066 Epalinges, Switzerland

## Abstract

**Background:**

To understand cancer-related modifications to transcriptional programs requires detailed knowledge about the activation of signal-transduction pathways and gene expression programs. To investigate the mechanisms of target gene regulation by human estrogen receptor α (hERα), we combine extensive location and expression datasets with genomic sequence analysis. In particular, we study the influence of patterns of DNA occupancy by hERα on expression phenotypes.

**Results:**

We find that strong ChIP-chip sites co-localize with strong hERα consensus sites and detect nucleotide bias near hERα sites. The localization of ChIP-chip sites relative to annotated genes shows that weak sites are enriched near transcription start sites, while stronger sites show no positional bias. Assessing the relationship between binding configurations and expression phenotypes, we find binding sites downstream of the transcription start site (TSS) to be equally good or better predictors of hERα-mediated expression as upstream sites. The study of FOX and SP1 cofactor sites near hERα ChIP sites shows that induced genes frequently have FOX or SP1 sites. Finally we integrate these multiple datasets to define a high confidence set of primary hERα target genes.

**Conclusion:**

Our results support the model of long-range interactions of hERα with the promoter-bound cofactor SP1 residing at the promoter of hERα target genes. FOX motifs co-occur with hERα motifs along responsive genes. Importantly we show that the spatial arrangement of sites near the start sites and within the full transcript is important in determining response to estrogen signaling.

## Background

Human estrogen receptor alpha (hERα) is an essential nuclear receptor regulating female development and reproductive functions. In the context of breast cancer, both hERα protein concentration and mRNA abundance have been shown to be associated with specific cancer sub-types and to influence survival rates [1-3]

Estrogen receptor is known to bind DNA at estrogen responsive elements (EREs) and to activate transcription of its target genes, in particular early estrogen-responsive genes [4-7]. It was also shown that some hERα targets, such as c-Myc, lack the ERE, but instead contain AP1 or SP1 binding sites, which appear to be essential for transcription *in vitro *[5,8,9]. Studies about how the estrogen receptor eventually induces transcription of its target genes have lead to the identification of complex interactions between hERα and a large number of cofactors [7,10,11].

Several recent approaches have been developed to identify hERα targets in vivo on a genome-wide scale. Correlation studies have used mRNA expression levels [12] from compendia of cancer samples to identify estrogen induced genes in cells. Other approaches have resulted in refined binding motifs from sequence analysis [13], novel candidate regulatory elements from comparative genomics, and most recently high-resolution maps of binding sites from ChIP-chip [14-16].

From ChIP-chip studies, Carroll and colleagues [15] drew the conclusion that estrogen receptor can activate transcription when bound to distal enhancers and that it is assisted in this function by the transcription factor FOXA1. The authors eventually refined their conclusions stating that FOXA1 can translate epigenetic signatures into cell-type specific transcriptional programs; i.e. FOXA1 recruits hERα, or androgen receptor, which, in turn, may act as stabilizer for FOXA1 binding [17]. Enrichment of FOXA1 binding seems to be most evident around intergenic ERE's, and almost undetectable at promoters [16].

Another approach uses a ChIP paired end diTags (ChIP-pet) technique to map hERα binding sites [18]. The authors found that the majority of hERα binding events happen in intragenic regions, in particular in introns; hERα sites at promoters are capable of inducing transcription, as are hERα binding sites in distal enhancers, as previously reported [15]. The ChIP-pet investigation [18] found poor conservation of estrogen receptor binding sites between human and mouse, and overlaps in the consensus motifs of hERα and putative transcription factor partners such as AP1.

A previous study [19] isolated 12 transcriptionally active genomic sites which recruit hERα. It showed one case of an ERE located 3.7 kb downstream of the first transcriptional start site of a target gene. Moreover, the presence of the SP1 transcription factor at the promoter of genes induced by hERα was shown by ChIP. Nevertheless, SP1 binding was not influenced by estradiol, pointing to independent mechanisms of recruitment. Further expression profiling analyses [20] tackled the same question employing an inhibitor of transcription, cycloheximide, to discriminate between primary and secondary targets. The authors concluded that AP1 and GC-box binding factors such as SP1, are enriched around the transcription start sites of up-regulated primary targets. Thus local nucleotide composition seems to play a role in defining active ERE's. This observation is supported by *in silico *investigations [13] reporting that extending the hERα PWM with CG rich flanks improves the prediction of functional hERα binding sites. Transcriptional regulation of hERα secondary target genes is, on the other hand, controlled by E2F, a transcription factor involved in cell-cycle regulation [20].

In this study we investigate mechanisms of target gene regulation by hERα by studying the relationship between EREs and gene expression. For this, we combine independent genome-wide ChIP analyses with large-scale microarray studies of estrogen response as well as genomic sequence analysis. In this context, we also investigate the roles of binding sites of known cofactors such as FOX and SP1 *in silico*. Our analysis confirms important characteristics of EREs such as their intergenic localization, the presence of cofactor sites, and GC biased local nucleotide composition. Unexpected however, was the finding that stronger and weaker hERα sites show different localization patterns with respect to annotated transcripts. Specifically, weak sites are enriched near transcription start sites, while stronger sites show no positional bias. We then study the relationship between binding and expression patterns and find binding sites downstream of the transcription start site to be equally good or better predictors of hERα-mediated expression than upstream sites. Studying FOX and SP1 cofactor sites near hERα ChIP sites shows that both factors reinforce the response to estrogen. Taken together, our results argue against significant hERα activity as a promoter bound transcription factor, and rather favor the long-range interaction model involving SP1 as the main mechanism of hERα-mediated response to estrogen. Finally we compile a reliable set of direct targets related to both normal and pathological states using our integrative approach.

## Results

### Strong ChIP-chip sites co-localize with strong hERα consensus sites

**Figure 1:**
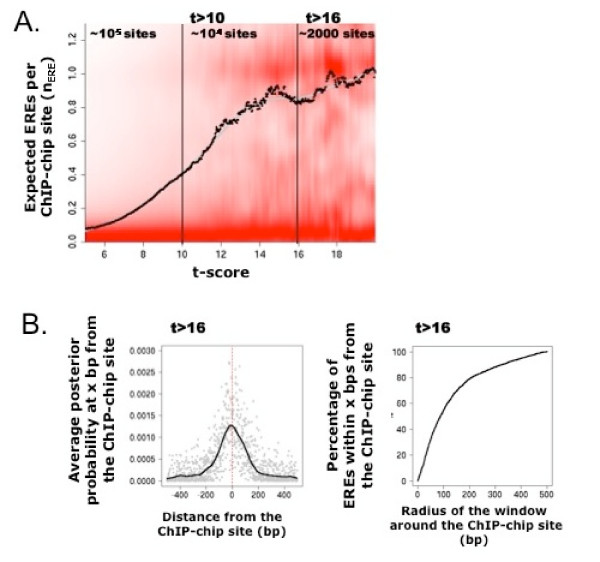
**EREs and ChIP sites**. **A**. Number of hERα sites for 1 kbp sequences centered around the ChIP sites identified by SLM. The number of sites is computed from a Hidden Markov Model (cf. Methods) using posterior decoding. Results are stratified in function of the strength of the binding site (t-score). The density profile (red) shows bimodality for high t-scores. The median (dots) is calculated in bins of one unit in t-scores. A smoothed estimator (in grey) has been added as visual aid. The cut-offs used for defining highest (t < 16) and lower stringency sites (t > 10) are indicated with vertical lines. The monotonous trend can be approximated by a sigmoid (tanh) function with half-height at t~10 and saturating at t~16 (>90%). **B**. Left: Average occupation profile at each genomic position computed using posterior decoding for the hERα consensus (e.g. 0.01 means that 1% of sequences have an ERE at this precise position). The profile is centered on the mode of the ChIP-chip site (red dashed line). Right: Fraction of EREs within a given radius of the mode of the ChIP signal. The ChIP sites identified with SLM have a width of about 1 kbp (width of the peak) while the binding sites for the 80% sites with a consensus (one position with posterior probability >0.5) are found within 200-bp of the mode in the t-profile.

We analyzed ChIP data for hERα measured by Affymetrix tiling array [15] using our signal estimation method SLM [21]. This method provides a quantitative measure (t-score) of the ChIP enrichment that allows strong and weak signals to be differentiated. As a first assessment of the fidelity of the binding regions, we measure the presence of canonical EREs (15 bp consensus motifs; cf. Methods) within 500 bp of mapped ChIP sites. For this, we designed a Hidden Markov Model (HMM) model for hERα binding, allowing multiple non-overlapping EREs per sequence (cf. Methods). Specifically, we compute posterior probability to find EREs at each position along the 1 kbp sequences centered on the sites identified by SLM. We observe that the expected occurrences of EREs show a monotone sigmoidal dependency on the corresponding t-scores (Fig. 1A). This behavior allows to define cutoffs for selecting high confidence ChIP sites in a natural manner: for t~16 each site has on average one ERE, which is double the number found for t~10. The actual cutoffs were determined from a sigmoidal fit to the median occurrence in function of t-scores (Fig. 1A). We thus define a set 2359 high stringency sites with t = 16 and a set of 7444 lower stringency with 10<t<16. The latter group could reflect weaker binding sites stabilized by the presence of co-factors, or give indications of cross-linking of long-range interactions between hERα and other factors [14]. Interestingly, we find that the genomic coordinates of EREs (posterior probability > 0.5) fall within a narrow window of the estimated positions of ChIP sites. Namely, 80% of EREs resides within 200 bp from the position of maximal ChIP enrichment as modeled in the SLM method (Fig. 1B and [see Additional file 1]). This means that although the resolution of ChIP is limited by the size of the fragments, typically about 1 kb, the position of the binding site occurs in 80% of cases within 200 bp of the maximum enrichment.

### Nucleotide bias near hERα sites

**Figure 2:**
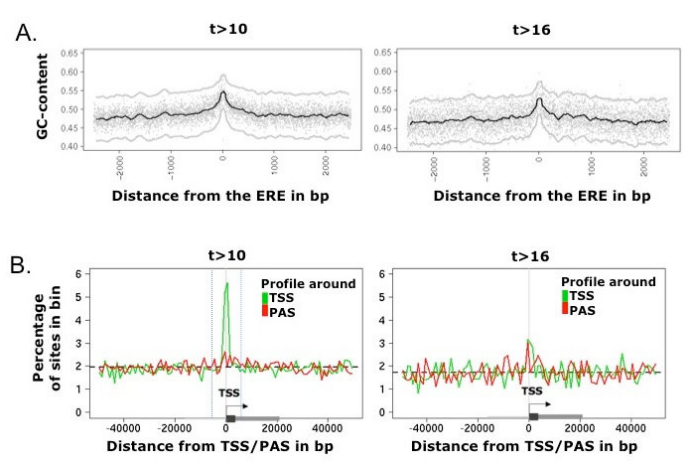
**Characteristics of weak and strong ChIP sites**. **A**. Average nucleotide composition profile for ChIP sites with ERE consensus sites (posterior probability > 0.5). The sequences are centered on the ERE. Both sets, the low (left panel) and high (right panel) stringency sites, show a maximum GC enrichment within 200 bp of the ERE. Notice that GC content has not reached genome wide baseline at +/- 2.5 kbp, and drop-off is faster for the stronger sites (right). Each gray dot represents the mean frequency at one position, smoothed mean (black) +/- 2SD (gray) and shown as lines. **B**. Localization of hERα binding sites relative to annotated transcription start sites (TSSs) and poly-adenylation sites (PASs). The percentage of occurrence is calculated relative to the number of sites in the full window (± 50 kbp of TSS or of PAS, bin size 500 bp). Coordinates are taken positive in the transcript direction but results show absence of directionality in the profiles. Left panel: Distribution of distances from TSSs for sites with 10<t<16 mapped in the 5' regions. The noticeable peak around the TSS covers 12% of the total number of sites in the region. We thus find a tight colocalization with the TSS (defined as 0, green profile) for a subset of sites. In contrast, no colocalization is evident for the PAS (red profile). Right panel: Distribution of distances from TSSs for sites with t>16 mapped in the 5' regions. In this case, sites are uniformly distributed in the 50 kbp around the TSS (green profile) and around the PAS (red profile).

Studying the nucleotide composition around hERα binding sites requires a precise mapping of the hERα motif on the genomic sequence, so that sites can be aligned with respect to EREs. We inspect all high and lower stringency sites with at least one ERE occurrence (posterior probability > 0.5) within 1 kbp of the reported ChIP-chip site. We find that the sequence at these sites is GC-rich (~46% GC) compared to the genomic background composition (~40%) and varies with t-score, the maximum GC-content being found for sites with t~10 (~50% GC, [see Additional file 2], panelB). Moreover, the average sequence composition exhibits an increase in GC-content of ~4% in 200 bp region immediately around the hERα binding site (Fig. 2A). We note that this is not due to the bias in the ERE itself (GC is 60%) but due to the flanking bases. In addition, within the studied window, the GC bias stays higher for the weaker sites, consistent with the higher frequency of weak sites near transcription start sites (cf. next paragraph).

### Localization of ChIP-chip sites relative to genome annotations

To further characterize hERα sites, we examine their localization relative to known genes. We use UCSC annotations (cf. Methods) and find that high stringency hERα binding sites (t>16) occur both in intergenic regions (~56%) and along genes (~44%) (Table 1). Interestingly, we observe an enrichment of low stringency sites (10<t<16) binding sites within 500 bp of transcription start sites (TSSs) which was not reported previously. In particular 12% of sites within 50 bkp of a TSS are closer than 500 bp to the TSS (Fig. 2B, left panel). In comparison, no detectable enrichment is present near the poly-adenylation site (PAS) (Fig. 2B, left panel). Finally, a remarkable fraction of sites, 16.7%, lies in repeats (cf. Table 1). This fraction is higher than the 5.3% reported by previous analysis of the ChIP-chip data [15], but not as high as the 27.9% reported by ChIP-pet [18].

**Table 1:** Number of hERα sites relative to genome annotations and repeats.

t-score > 16	Total	In genes	Intergenic	In repeats
Number of sites	2359	1041	1318	396

**Columns 1–3**: Detected sites and their positions with respect to annotated transcripts

(UCSC genome browser, hg18).

**Column 4**: Detected sites residing in regions marked as repeats by RepeatMasker as reported in UCSC genome browser tables.

To be associated with a transcript, a site is required to lie between -5 kb and +5 kb of the annotated boundaries (transcription start site and poly-adenylation site). A complete list is available upon request to the authors. The smoothing used in SLM is σ = 200 bp [21].

### The number of binding sites downstream of the TSS is a good indicator of hERα-mediated induction

To investigate how the occupation of hERα binding sites leads to gene induction, we assess the influence of hERα binding on expression phenotypes. The assessment requires appropriate assignment of a binding site to the target gene: generally a binding site is considered to influence the expression of a gene if it lies within a predefined window around the TSS or the gene. Previous studies [7,14-19] have used different windows: tight cis-regulatory regions upstream of TSS [16]; 50 kbp windows around the promoters of responsive genes [15]; 100 kbp around the promoters of cancer related genes [18]. Here we assess which definition is best at discriminating induced genes. For this, we quantify the expression response to estrogen for each transcript using four independent datasets probing different aspects of estrogen signaling (cf. Methods). We will focus mainly on two datasets: an expression compendium of cancer tissue samples [12] and a study on MCF7 cells where secondary target activation has been blocked by addition of cycloheximide, an inhibitor of protein synthesis [20]. For breast cancer samples, we expect that primary targets of ESR1 will correlate with ESR1 mRNA abundance as protein concentration and mRNA abundance have been shown to associate with cancer sub-types [12] (cf. linear model, methods). Taken together, the different experimental settings give us a broad picture of the activity of hERα and its targets. We compare the various assignments of sites using receiver operating characteristic (ROC) analysis, a commonly used methodology to assess the tradeoff between the sensitivity and specificity of predictors.

**Figure 3:**
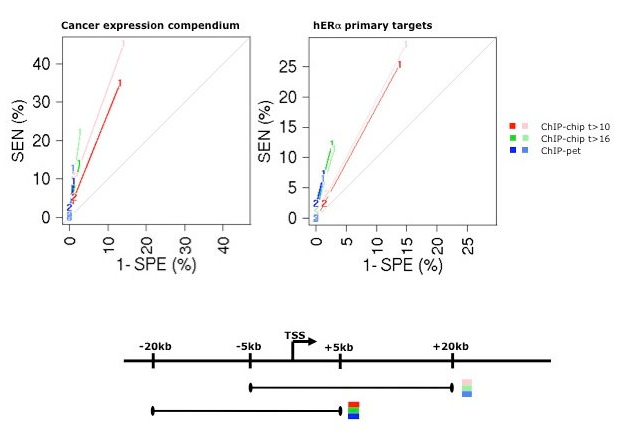
**ROC analysis for comparing the ability of upstream or downstream ChIP sites to predict induced genes**. In each experiment, the induced genes (positives) are taken as the 1% highest ranking transcripts. The remaining 99% are taken as the negatives. Note that for discrete data such as the number of sites in a specific genomic window, ROC analysis consists in a set of operative points (cf. Methods). The number of sites downstream of the TSS (light curves) shows the best performance among all the definitions tested (high stringency and low stringency ChIP-chip, ChIP-pet). Shown are the cancer expression compendium [12] and the study on primary estrogen receptor targets [20]. Further expression sets are shown in [see Additional file 3].

We first compare the performance of proximal and upstream sites vs. proximal and downstream binding sites. We find that the number of binding sites covering the 20 kbp downstream of the TSS tend to be a better discriminator than the number in the equivalent upstream region. Though the ROC curves show little differences, the trend suggests that downstream sites yield higher sensitivity at almost identical specificity for each operative point (Fig. 3). Different sets of binding sites, i.e. high, low stringency ChIP sites, or ChIP-pet sites [18], show slightly altered sensitivity and specificity, but the downstream sites perform consistently better than upstream sites (Fig. 3 and [see Additional file 3]). We remark that few genes have more than one site within 20 kbp of the TSS, thus resulting in low sensitivity at high specificity; however, the set of genes with more than one site is highly enriched for direct targets, the enrichment being the slope of the line connecting the operative point to the origin.

**Figure 4:**
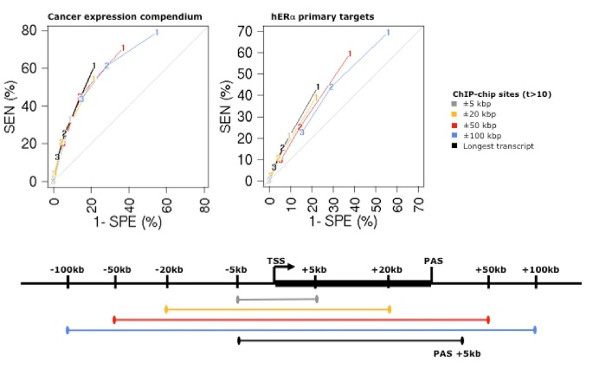
**ROC analysis to compare the ability of ChIP sites in variably sized windows to predict induced genes**. Positives and negatives are taken as in Fig. 3. Number and definition of operative points is as in Fig. 3. The discrete curves lie on the same envelope, but the number of sites along the transcript (black curve) shows the best performance for all the sites definitions used (high stringency and low stringency ChIP-chip, ChIP-pet). The expression datasets are the cancer expression compendium and the study on primary estrogen receptor targets. In each experiment, the induced genes (positives) are taken as the 1% highest ranking transcripts. The remaining 99% are taken as the negatives. The same analyses using different definitions of sites (high stringency or ChIP-pet) are given in [see Additional file 4]. Further expression sets are analyzed in [see Additional file 5].

Secondly, we compare definitions with varying regulatory region sizes. We use the number of binding sites in the region as a discriminator for estrogen sensitive genes (Fig. 4, [see Additional file 4] and [see Additional file 5]) and introduce the number of binding sites along the transcript as a new metric for hERα-mediated gene induction. This definition refers to sites along the whole length of the transcript from 5 kbp upstream of the transcription start site to 5 kbp downstream of the poly-adenylation site. We observe that the operative points for the different definitions lie on the same envelope, bounded on the left side by the ROC curve for the number of binding sites along the transcript (Fig. 4, [see Additional file 4] and [see Additional file 5]). At equal sensitivity, the number of sites along the transcript achieves comparable or better specificity (Fig. 4, black curve) than the other definitions in both expression datasets. Taken together, these results show that ERα sites located downstream of start sites are equally or more effective at inducing genes than upstream sites.

**Figure 5:**
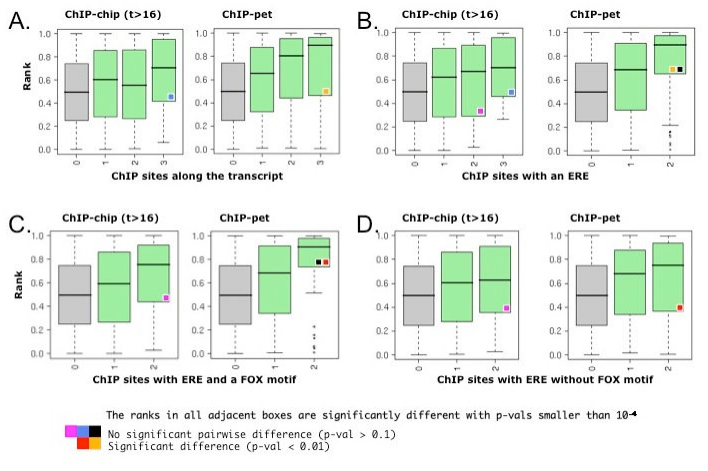
**Response to hERα increases in function of the number of ChIP sites along transcripts**. Ranks of the induction scores are shown as boxplots in function of the number of ChIP sites. In **B-D**, ChIP sites are further filtered according to the presence or absence of consensus elements for hERα (**A**) or FOX (**C, D**). A motif is assigned to a binding site if the occupancy, computed using posterior decoding, is greater than 0.5 (cf. Methods). The ranks of the induction scores of the cancer expression compendium and of the study on primary estrogen receptor targets have been pooled to avoid small sample size effects. Significance of the comparisons is assessed using the Wilcoxon rank sum test, i.e. comparison between equally colored distributions are made. **A-B**. Effect of ERE motifs. For ChIP-pet sites the presence of an ERE improves the correlation between ranks and number of sites (orange boxes). No statistically significant improvement is detected for the ChIP-chip sites (blue boxes). **C-D**. Effect of FOX motifs. In addition to the presence of an ERE, the presence of a FOX motif improves significantly the association for the ChIP-pet sites (red box). Comparison with panel B (black boxes) indicates that many ChIP-pet sites with EREs also have FOX sites. Despite a shift in the distribution to higher ranks, no statistically significant improvement is detected for the ChIP-chip sites (purple boxes) with a FOX site.

In a complementary analysis, we study the ranks of induction in function of the number of hERα sites along transcripts. Here the ranks are taken as the merged ranks from the cancer expression compendium and primary target datasets. We find that the ranks clearly correlate positively with the number of sites both for the ChIP-chip and ChIP-pet sites. The latter shows a more pronounced effect indicating that ChIP-pet sites occur with preference near strongly induced targets (Fig. 5A). The positive correlation is generally unchanged when we restrict the sites to those harboring a good instance of an ERE, but note that it is increased in case of two ChIP-pet sites (Fig. 5B). Thus sites with EREs are only marginally more prone to lead to increased transcriptional response.

### Signatures of FOX and SP1 cofactor sites near hERα ChIP sites

Though the ERE is the dominant recognition sequence for strong ChIP sites (t>16), other transcription factors are also involved in the regulation of target genes by direct interaction with hERα at its specific sites [14,17,19]. We test the contribution to gene induction by analyzing the presence or absence of co-factor sites. We map the cofactors sites based on their consensus sites by extending the cyclic HMM model to reported co-factors of hERα: FOXA1, AP1 and SP1 (cf. Methods, [see Additional file 1]). We also include E2F as this factor is known to regulate secondary targets of the hERα signaling cascade [20]. We find the SP1 consensus to be most represented in low stringency ChIP sites (10<t<16), and observe a concurrent low expectation for FOXA1 sites. For high stringency sequences (t>16), these two signals reach similar occurrences (a median of 0.7 sites) and appear to follow a similar slowly increasing trend as the EREs ([see Additional file 1], red and green profiles). At the stringencies implemented in our HMM, AP1 occurrences are uniform over the full range of t-scores while E2F motifs are rare ([see Additional file 1], orange profile), consistent with the role of E2F in the induction of secondary targets [20]. We find similar spatial profiles for the cofactor sites as in [15], *i.e*. a bias towards the position of the maximal ChIP-chip signal for the EREs (Fig. 1B), for FOXA1 sites and for AP1 sites (not shown). In further characterizing the FOXA1 sites, we find no difference between the log-likelihood of the FOXA1 in the high-stringency sites and the log-likelihood of other motifs from the FOX factors [see Additional file 6]. The observed signal is thus to be attributed to a generic FOX site rather than specifically to FOXA1.

### Induced genes have ChIP sites with FOX or SP1 sites

**Figure 6:**
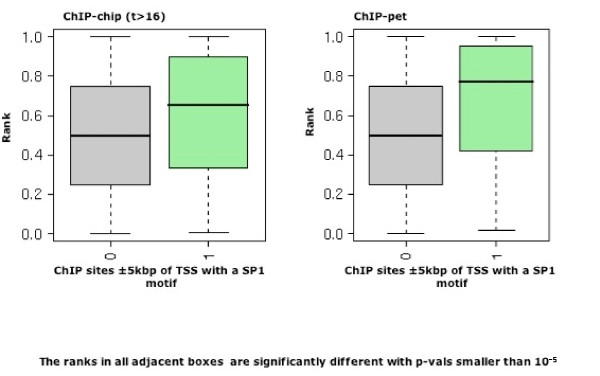
**SP1 acts as a cofactor for promoter proximal hERα sites**. Ranks of the induction scores are shown as boxplots in function of the number of promoter proximal (± 5 kbp around the TSS) ChIP sites with and without SP1 sites. The presence of SP1 motifs underlying promoter proximal ChIP sites increases the induction rank. Ranks are computed as in Fig. 5 and SP1 motifs are assigned to a ChIP site if the occupancy is greater than 0.5 (Cf. Methods). Significance is assessed by Wilcoxon rank sum test.

In order to study the dependency of hERα mediated induction on co-factors, we stratify the ChIP sites along the transcript according to the presence of FOX and SP1. FOX has been reported to be involved in the regulation of hERα target genes, in particular through remodeling of chromatin prior to hERα recruitment [14,15,17]. On the other hand, SP1 is a GC-box bound enhancer associated with the activity of the transcription machinery [22,23] and frequently localized in the proximity of TSSs [21,24]. As expected, we find a strong enrichment of SP1 sites for sites near promoters, compared to GC-rich binding sites located in non-promoter proximal regions ([see Additional file 2], panels C-D). The analysis of hERα sites with EREs, stratified according to FOX sites, shows that the correlation with induction is preserved independently of FOX sites. However, the presence of FOX sites shifts the distribution toward higher induction in the case of the ChIP-pet sites (p-val < 0.01, rank test, Figs. 5C–D, red square). Separately we also consider hERα ChIP sites around the TSSs harboring GC-boxes (SP1 sites). Clearly, the set of targets with one binding site harboring a GC-box is strikingly enriched for induced genes, both in the case of ChIP-chip mapping and in the case of ChIP-pet mapping (Fig. 6 and fifth column of [see Additional file 7]). Together, the analysis of putative cofactor sites is consistent with an activating role for the FOX factor next to EREs, while the role of SP1 sites in mediating response to estrogen at promoter proximal ChIP sites is very clear, irrespective of EREs.

### High confidence hERα target genes

As reported targets tend to differ across experiments [7], we used these multiple datasets to identify 35 genes with at least two high-stringency ChIP-chip sites, one ChIP-pet site along their full-length transcript and ranking in the top 10% of hERα responding genes (Table 2). 12 of these genes have previously been reported as direct targets of hERα [14,15,18,19] and they have been associated with the transduction of estrogen signaling activity. All of these 12 direct targets show the presence of at least one ERE along their transcript, often accompanied by one or more FOX motifs. Three known targets also exhibit SP1 motifs in their promoter regions (Table 2, 4^th ^column).

**Table 2:** List of candidate targets of hERα.

Gene symbol	EREs	FOX	SP1	Association to ESR1
AFF3	1	1	0	Not yet reported
ANXA9	1	0	1	Not yet reported
ARL3	1	1	0	Not yet reported
ARSG	0	0	0	Estrogen signaling
ATRNL1	0	1	0	Not yet reported
C6orf97	1	1	0	Upstream of ESR1, no association reported
CELSR1	2	0	0	Cancer signature
CISH	1	0	0	Known target of ESR1
CXXC5	3	2	1	Not yet reported
CYP24A1	0	0	0	Breast cancer signature
DSCAM	2	3	0	Known target of ESR1
ERBB4	1	2	0	Estrogen signaling
FAM63A	1	0	1	Not yet reported
FSIP1	1	1	0	Not yet reported
GREB1	1	0	1	Known target of ESR1
IGF1R	1	2	0	Known target of ESR1
JMJD2B	2	1	1	Known target of ESR1
LMBR1	1	1	0	Anterior upper body development
MLPH	2	2	1	Anterior upper body development
MYB	1	1	0	Known target of ESR1
PARD6B	0	1	0	Known target of ESR1
PDZK1	2	3	0	Known target of ESR1
PKIB	2	2	0	Not yet reported
PREX1	2	1	0	Cancer signature
PTPRG	2	4	0	Known target of ESR1
RARA	1	0	0	Known target of ESR1
SFXN2	1	1	0	Not yet reported
SGK3	1	1	1	Cancer signature
SIAH2	2	2	0	Known target of ESR1
SLC22A5	1	1	0	Cancer signature
SLC9A3R1	3	1	0	Cancer signature
SPOCK1	1	1	0	Cancer signature
STARD10	1	1	0	Breast cancer signature
TFF1	1	1	1	Known target of ESR1
VAV3	2	3	0	Estrogen signaling

Each selected target shows at least two high stringency ChIP-chip sites and one ChIP-pet site along its transcript. In addition it scores in the 10% highest ranks in the combined rank list (cf. Fig. 5).

**Column 1**: Gene symbol. (UCSC genome browser, hg18).

**Column 2–4**: Number of binding motifs for hERα (EREs), FOX and SP1 respectively. EREs and FOX motifs are counted along the entire transcript, SP1 motifs are counted in ± 5 kbp of the TSS.

**Column 5**: Brief annotation of the known association of the target to estrogen activity, or to cancer. Breast cancer signature and cancer signature reflect upregulation of the corresponding transcript in one of the reported conditions.

Of the remaining 23 genes, 3 genes (*arsg*, *erbb4 *and *vav3*) are part of the estrogen signaling pathway, and two are involved in the development of the upper body: with the only exception of *arsg*, these genes exhibit a high number of EREs and of FOX sites (Table 2, 2^nd ^and 3^rd ^columns). Eight genes are associated with cancer [18,19], or appeared upregulated in previous cancer studies [25-27]. Two of them (*cyp24a1 *and *stard10*) are specifically associated with breast cancer [25-27]. We observe that these genes tend to have both EREs and FOX sites, but no clear SP1 site at promoters. In addition, the number of EREs seems to be higher than the number of FOX sites. Except for *c6orf97*, a 127 kbp long open reading frame located upstream of the *esr1 *gene, the remaining ten hERα targets have not yet been associated to estrogen signaling or cancer activity. Though it is unclear how these targets may be implicated in estrogen signaling, we find that *anxa9, arl3 *and *atrnl1 *are recruited during vesicle trafficking and signaling, probably performing relevant functions downstream of the estrogen response. Further experiments will be needed to clarify their role downstream of hERα.

## Discussion

### Properties of hERα binding sites measured in ChIP experiments

We analyzed raw genome-wide ChIP-chip data [15] using our previously developed SLM algorithm [21]. One important advantage of this method is the ability to rank sites according to their strength (t-score). This gives an approximate quantification of the residency time, and allows us to stratify our analyses accordingly. The procedure has highlighted an interesting difference in the localization patterns between low and high stringency sites: low stringency sites are enriched in close proximity to TSSs, while strong sites show no bias in localization as previously discussed [15,28]. Expectedly we find that the number of predicted EREs increases with the stringency of the observed sites, with 50% of highest stringency sites showing a good match to a full ERE. This is less than the 71% reported in a Chip-pet study [18] which used a comparable definition of consensus match. This difference is in line with the notion that ChIP-pet sites are enriched in higher stringency sites as compared to the ChIP-chip sites [15]. Finally we describe a nucleotide composition profile showing a peak in GC bias within a narrow window (~200 bp) around the EREs; we note that this effect is uncoupled from CpG islands as for t>16 only 5% (66 sites) of the sites considered overlap with CpG islands (10% for t>10) and the GC bias is unchanged if these are removed. Our findings are in line with previous bioinformatics analyses [29] which showed that regulatory regions have a biased nucleotide composition compared to the rest of the genome, in particular showing higher GC content than expected. In addition, [13] stated that EREs with CG-rich flanks are better predictors of functional regulatory binding sites. Considering that these authors performed an unbiased search of the genome, their result matches our observation that both low- and high-stringency ChIP-chip sites are enriched in CG-content relative to the background (t<10).

### Assigning hERα binding sites to target transcripts

Linking ChIP-chip sites to expression phenotypes requires mapping of sites to transcripts. This is a notoriously difficult problem in higher eukaryotic organisms due to the potential long-range regulation from distal enhancers. In the absence of a better solution, the current practice is to assign a site to a TSS according to proximity, using *ad hoc *window sizes, both symmetrically and non-symmetrically centered around TSSs. We assessed a variety of proposed definitions in their ability to predict expression status of target genes and found downstream sites to be equally good or better predictors of hERα-mediated expression than upstream sites. While it has been reported that ChIP sites tend to be symmetrically distributed around TSSs for a variety of transcription factors [30], it is likely that the increased performance of downstream sites is due to fewer ambiguous assignments to transcripts when genes are closely spaced.

### Functional signatures of cofactor elements near hERα sites

Several transcription regulators and chromatin remodeling factors have been shown to interact with hERα. Recently, [15] demonstrated that hERα and FOXA1 interact over long ranges while [17] argued that hERα and FOXA1 may reciprocally help each other in stabilizing binding to DNA. Moreover ERE independent gene activation relying on hERα/SP1 complexes at GC-rich sites has been reported [31]. We therefore used our metrics to study the influence of FOX and SP1 cofactor sites near hERα ChIP sites on the induction of target genes. Consistent with the aforementioned hERα/SP1 mode of regulation we find that promoter proximal sites are much more effective at inducing expression when they co-occur with an SP1 site.

### Two models for hERα target regulation

Taken together, these data suggest two hypotheses: (i) weak DNA interactions, in particular at promoters, represent degenerate EREs bound by hERα and compensated by the presence of cofactors; (ii) alternatively, these weak ChIP signals are the result of cross-linking of long-range interaction of hERα with its promoter-bound partners. Both scenarios are consistent with our analysis. In support of the first we showed that promoter proximal ChIP binding sites, i.e. in centered windows of 5 kbp, are associated with gene induction (Fig. 4). Moreover, genes with SP1 motifs in their promoter sequences show strong estrogenic response independently of EREs (Fig. 6). However, our finding that consideration of downstream sites far from promoters increases the selection of induced genes would favor the second model in which hERα-mediated induction of target genes is more prominently regulated through long-range interactions. Indeed, we find that the number of ChIP sites along the transcript is the best discriminator for highly responsive genes (Fig. 5 and [see Additional file 7]). In this analysis, the occurrence of cofactor motifs, i.e. FOX, only marginally improves the discrimination of highly induced target genes (Fig. 5).

### Data integration and high confidence hERα targets

An admitted caveat of independent location and expression studies is that the list of predicted targets can be quite large [20]. Crossing results from several studies will likely form a core group of the most robust targets. In this context, we applied stringent criteria to define a list of high confidence direct targets. We found that there was a distinction between targets associated to normal conditions and targets which are most prominent in pathological states. Namely, known targets and targets related to estrogen signaling and fore-body development are rich in both EREs and FOX motifs, but they also show the presence of SP1 sites (Table 2). On the other hand, targets that have been reported in cancer studies, probably reflecting abnormal hERα activity at binding sites, exhibit many EREs but no SP1 motifs at promoters and few FOX sites along the transcripts (Table 2).

## Conclusion

We investigated the influence of hERα binding patterns, as detected by ChIP, on expression phenotypes in cell culture and cancer tissues, measured by microarray. Furthermore, the availability of a ChIP-chip dataset [15] and a ChIP-pet dataset [18] enabled the direct comparison of the two technologies. ChIP-chip being sensitive to cross-linking, the methodology can be informative in detecting weaker or transient enrichment at promoters. The present study highlights how such weak sites show different functional signatures from the stronger sites. ChIP-pet, on the other hand, selects stronger sites and offers higher positional resolution. Localizing binding elements *in silico *using genomic sequence analysis in conjunction with ChIP helps stratify sites and highlight relevant features underlying induction by hER.

Taken together, our analyses support the model that hERα mediated response to estrogen signaling occurs over long-range interactions with SP1 or other promoter-proximal cofactors. The strength of the response is in quantitative linkage with the number of hERα binding sites along target transcripts. We found that the intragenic fraction of bound hERα is the best discriminator of estrogen responsive genes. In particular, our main finding is that the number of hERα binding sites along the transcripts of target genes is correlated with the strength of the response. This highlights the quantitative nature of the hERα-mediated response to estrogen.

## Methods

### Genomic data

Genomic sequence, annotations and chromosomal coordinates of transcription start sites (TSSs) are publicly available from the UCSC Genome Table browser [32]. Based on these coordinates, we define "genes" as the genomic regions from 5 kb upstream of the TSS to 5 kb downstream of the polyadenylation site (PAS), accounting for roughly 30% of the chromosomes length. We use the genome build hg18.

### ChIP data

Shirley Liu provided the raw ChIP-chip data upon request [15].

In brief, estrogen receptor proteins were cross-linked to DNA and purified using specific antibodies. Fragments were amplified with random primers and hybridized on Affymetrix Human tiling arrays, covering the non-repetitive genomic sequences of the human genome. The data provides three technical replicas [15]. To quantify the enrichment we used our SLM algorithm [21] without applying the resampling technique to control the false discovery proportion.

The coordinates of the ChIP-pet study are publicly available online [18].

### Expression microarray data and induction scores

The compendium of cancer expression was used to derive the partial correlations of every gene in the genome, as in the following

Where  is the expression level of gene *g *in condition *i*; *α*, *β*, *γ*, *δ *and *λ *are the partial correlations. The genes ESR1, ERB2, AURRA, PLAU and STAT1 correspond to estrogen pathway, 17q amplification, proliferation, stroma and immune respone, respectively.

The method is a linear model, fitted separatedly for each dataset in [12]. Each term produces a partial (adjusted) correlation. The partial correlations are combined across datasets using the Fisher's hyperbolic tangent formula. The partial correlation to ESR1, *α*, has been used in the present study.

The primary hERα target study is publicly available [20]; it consists of triplicates of 4 conditions: 2 conditions correspond to the estrogen induction (ER+) with and without cycloheximide (CHX+ and CHX-), and 2 conditions correspond to a mock induction non-estrogen related (ER-), with and without cycloheximide. We build two regression models to describe gene expression levels:(M1)(M2)

where *i *is the condition index, *I*_*g *_is a gene-dependent intercept, *a*_*g*_, *b*_*g*_, *a'*_*g *_and *b'*_*g *_are the regression coefficients, *δ *_*i *_is an indicator function taking value 1 when the condition expressed in *i *is satisfied, 0 otherwise. We reason that the M1 model is suited for direct targets, therefore, since cycloheximide inhibits expression of secondary targets, the inequality *a*_*g *_<*a'*_*g *_will be true for indirect targets. The induction score is the rank of the difference between *a*_*g *_and *a'*_*g*_.

We also consider other two publicly available MCF7 studies [15,33].

The estrogen dosage study [33] consists of 5 replicates of 5 conditions corresponding to the following concentrations of estrogen: 0 pM, 10 pM, 30 pM, 60 pM and 100 pM. We model the expression level of gene g according to the following linear regression model:(M3)

where *I*_*g *_is a gene-dependent intercept, *a*_*g *_and *b*_*g *_are the regression coefficients, *M *is a metagene averaging the expression levels of known responsive target genes of hERα (*tff1, rara, slc25a36, ddef2, wfikkn2, bcr*) [19] and *P *is a metagene averaging the expression levels of proliferation related genes (*vcy, pry, fam127a, ine1, serf1a, qser1, hbg1, opn1mw, akap2*) [34]. *a*_*g *_is the induction score.

The estrogen exposure study [15] consists of triplicates of 4 time points at which the population of MCF7 cells has been sampled and hybridized on the microarray: 0 h, 3 h, 6 h and 12 h. We model the expression levels of each gene similarly as in the estrogen dosage study. *a*_*g *_is the induction score.

Multilinear regression parameters and statistics are computed using the software R http://cran.r-project.org.

### Transcription factor position weight matrices (PWMs)

Position weight matrices (PWMs) were used to define consensus sites for transcription factors. To compute occupancies, PWMs were then embedded in Hidden Markov Models. The TRANSFAC [35] hERα PWM matrix, 19 bases wide, is poorly polarized for the first half of the dimer ([see Additional file 8], panel A), thus corresponding to an information content of 10.3 bits. By comparison, the TRANSFAC human E-box matrix, only 10 bases wide, carries 8.8 bits of information. In order to improve the ERE signal within the hERα PWM we recovered a de novo hERα PWM from the ChIP-chip data. Specifically we collected the ChIP-chip sites with t>16, and we analyzed the 250 underlying sequences that best matched the TRANSFAC matrix with MEME [36]. MEME identified a hERα motif as the strongest signal in our collection of sequences. We generated our de novo hERα PWM from the nucleotide composition of the sites recovered by MEME. A pseudocount of 1 nucleotide per position is included. Our hERα PWM matrix has an information content of 16 bits ([see Additional file 8], panel B, logo and frequency matrix).

The other PWM matrices used in the study are available on TRANSFAC [35]. When multiple matrices are available, we consider the one with the largest information content. The used matrices are: HNF3ALPHA_Q6_V_M00724 for FOXA1, E2F1_Q4_01_V_M00939 for E2F, AP1_Q6_01_V_M00925 for AP1 and SP1_Q6_01_M00196 for SP1.

### Hidden Markov Models (HMMs)

To infer a probabilistic segmentation of a sequence in terms of background and non-overlapping binding sites for a given set of transcription factors, we design a standard Hidden Markov Model (HMM). The emission frequencies for binding sites are taken from the aforementioned PWMs. The emission frequencies for the background state (0-order) are taken as the total genomic frequencies in human. We use a custom posterior decoding algorithm [37] to calculate the occupancy *n *at each site for each of the factors PWM. The graph of the multiple PWMs Markov chain of the hidden states implemented in the study is depicted in [see Additional file 1].

### ROC analysis

In the ROC analysis we compare discriminators (e.g. the number of sites along the transcript) to response variables (e.g. induction score measured on expression microarrays). We consider the 1% of most induced genes as genuine estrogen responsive genes. For each discriminator, we apply a sliding cutoff *x *to define a set of predicted positives P with *X>x*. The set of true positives, TP, corresponds to the elements in P which are also genuine estrogen responsive genes. Similarly, the set of false positives, FP, corresponds to the fraction of P which are not in the 1% of most induced genes. The set of negatives, N is the complementary to P. The false negatives, FN, are defined as the elements of N which are in the top 1% of most induced genes. The true negatives, TN, can be easily derived as TN = N-FN. We define sensitivity as the ratio TP/(TP+FN). Specificity is defined as TN/(FP+TN).

## Abbreviations

hERα: human estrogen receptor α; SP1: specificity factor 1; ERE: estrogen responsive elements; TSS: transcription start site; ChIP: chromatin-immunoprecipitation; ChIP-pet: ChIP paired end diTags; HMM: Hidden Markov Model; PAS: poly-adenylation site; ROC: receiver operating characteristic; PWMs: Position weight matrices; P: positives; N: negatives; TP: true positives; TN: true negatives; FP: false positives; FN: false negatives.

## Authors' contributions

FP carried out the ChIP-chip analysis, performed the statistical comparisons between the ChIP-chip and expression datasets. BS performed the alignment of the predicted binding sites to the human genome. PW and MD carried out the statistical analysis of the cancer expression compendium. FN designed and coordinated the study. FP and FN wrote the manuscript. All authors read and approved the final manuscript.

## Supplementary Material

Additional file 1 **Supplemental Figure S1**. Number of expected hERα and co-factor sites for 1 kbp sequences centered around the ChIP sites identified by SLM Click here for file

Additional file 2 **Supplemental Figure S2**. Sequence characteristics of ERE sites. Click here for file

Additional file 3 **Supplemental Figure S3**. ROC analysis for comparing the ability of upstream or downstream ChIP sites to predict induced genes. Click here for file

Additional file 4 **Supplemental Figure S4**. ROC analysis to compare the ability of ChIP sites in variably sized windows to predict induced genes: cancer expression compendium and primary targets. Click here for file

Additional file 5 **Supplemental Figure S5**. ROC analysis to compare the ability of ChIP sites in variably sized windows to predict induced genes: estrogen exposure and dosage datasets. Click here for file

Additional file 6 **Supplemental Figure S6**. Comparison of log-likelihood distributions of the FOX factors PWMs for the ChIP sites with t>16. Click here for file

Additional file 7 **Supplemental Figure S7**. Response to hERα increases in function of the number of ChIP sites along transcripts: only primary target dataset. Click here for file

Additional file 8 **Supplemental Figure S8**. Sequence logos for hERα position weight matrices (PWMs). Click here for file
