# Supplementary material for: Relationship between estrogen receptor α location and gene induction reveals the importance of downstream sites and cofactors
Source: BMC Genomics. 2009 Aug 18;10:381. doi: 10.1186/1471-2164-10-381 (PMC2907696; doi:10.1186/1471-2164-10-381)
Supplement: Additional file 1 — Supplemental Figure S1. Number of expected hERα and co-factor sites for 1 kbp sequences centered around the ChIP sites identified by SLM [file 1471-2164-10-381-S1.pdf]

Supplemental Figure S1

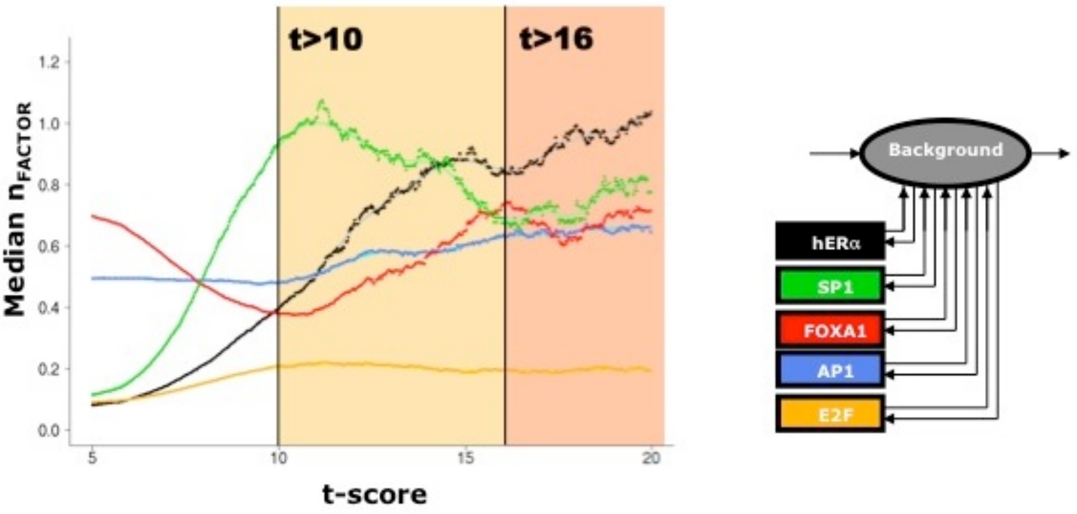

Fig. S1. Number of expected hERα and co-factor sites for 1kbp sequences centered around the ChIP sites identified by SLM.

Left Panel. Number of sites is computed from a Hidden Markov Model (cf. Methods) using posterior decoding. Results are stratified in function of the strength of the binding site (t-score). Factors are hERα (black), SP1 (green), FOXA1 (red), AP1 (blue) and E2F (orange) occupancy. At low t-scores, the AT-rich PWM for FOXA1 is favored by AT-rich sequences (Fig. S2B). Similarly the GC rich SP1 PWM is favored at t~10. For t>16 all profiles follow a saturating trend similar to hERα. The only exception is E2F, a regulator of hERα secondary targets (Bourdeau, et al.): as expected, E2F shows low occupancies of hERα ChIP-chip sites. The medians are calculated in bins of units of t-scores. Smoothing (lowess) estimators have been added for visual aid.

Right Panel. Scheme of the Markov chain of hidden states for the implemented HMMs. Each arrow represents a possible transition. The HMM allows transitions from background to hERα and cofactor PWMs. Reverse complements of the PWMs are not displayed for clarity. The transition probabilities from background to any of the co-factors PWMs are taken to be 2^{-11}.
