# Supplementary material for: Relationship between estrogen receptor α location and gene induction reveals the importance of downstream sites and cofactors
Source: BMC Genomics. 2009 Aug 18;10:381. doi: 10.1186/1471-2164-10-381 (PMC2907696; doi:10.1186/1471-2164-10-381)
Supplement: Additional file 2 — Supplemental Figure S2. Sequence characteristics of ERE sites. [file 1471-2164-10-381-S2.pdf]

Supplemental Figure S2

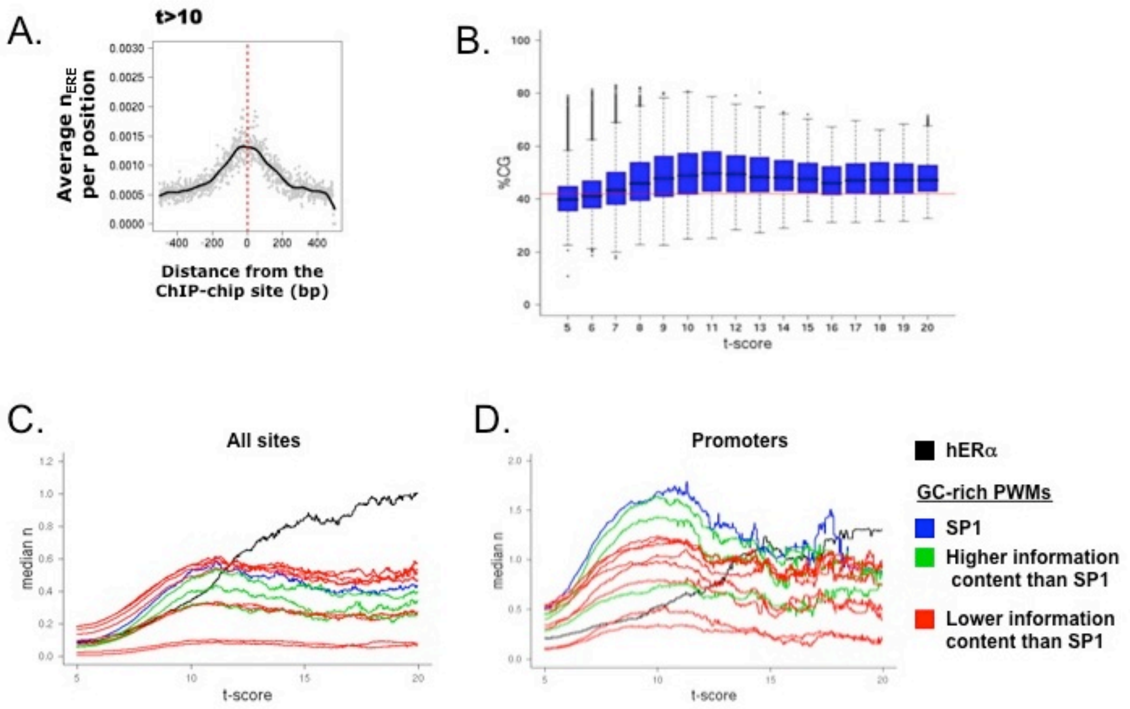

Fig. S2. Sequence characteristics of ERE sites.

A. Average nucleotide composition profile for ChIP sites with ERE consensus sites (posterior probability > 0.5). Idem as Fig. 2 but for the low stringency sites.

B. GC content (shown as boxplots) for the 400bp around the hERα ChIP sites. Bins cover one t-score unit. The sequences of ChIP sites with t>9 show enriched GC content over the background expectation (red line). The maximum GC-content enrichment over background is achieved at t~11. Median GC-content (thick horizontal bar in the box) for high confidence sites (t>16) is 50%.

C. Number of expected hERα and GC-rich factor sites for 1kbp sequences centered around the ChIP sites identified by SLM (similar to Fig.S1). Number of sites is computed from a Hidden Markov Model (cf. Methods) using posterior decoding. Results are stratified in function of the strength of the binding site (t-score). Besides hERa (in black) and SP1 (in blue), the factors are (with the respective TRANSFAC matrix and information content): E2F (E2F_Q2_V_M00803, 6.94 bits), ZNF219 (ZNF219_01_V_M01122, 15.54 bits), KROX (KROX_Q6_V_M00982, 12.45 bits), AP2g (AP2GAMMA_01_V_M00470, 8.30 bits), ETF (ETF_Q6_V_M00695, 7.90 bits), AP2 (AP2_Q6_01_V_M00915, 8.99 bits), MAZR (MAZR_01_V_M00491, 13.14 bits), AP2a (AP2ALPHA_01_V_M00469, 7.65 bits), AP2 (AP2_Q6_V_M00189, 8,40 bits), WT1 (WT1_Q6_V_M01118, 6.96 bits), MAZ (MAZ_Q6_V_M00649, 9.82 bits); colors have been assigned according to the information content: green for the PWM with higher information content than SP1 PWM, red for the others. The number of expected sites for CG-rich factors is correlated: however, SP1 competes with unspecific PWMs, showing a higher expected number of sites than more specific PWMs.

D. We used the same HMM model described in Fig.S2C, but we restricted the analysis to ChIP-chip sites in promoter proximal region defined as the 5kbp upstream of the TSS in the direction of the transcript. SP1 is clearly the factor with the largest number of expected sites in this regions with a peak also at t>16 which seem to be occurring for SP1 only. Unspecific PWMs (in red) generally score worse than the high specific ones (in green).
