# Supplementary material for: Relationship between estrogen receptor α location and gene induction reveals the importance of downstream sites and cofactors
Source: BMC Genomics. 2009 Aug 18;10:381. doi: 10.1186/1471-2164-10-381 (PMC2907696; doi:10.1186/1471-2164-10-381)
Supplement: Additional file 3 — Supplemental Figure S3. ROC analysis for comparing the ability of upstream or downstream ChIP sites to predict induced genes. [file 1471-2164-10-381-S3.pdf]

Supplemental Figure S3

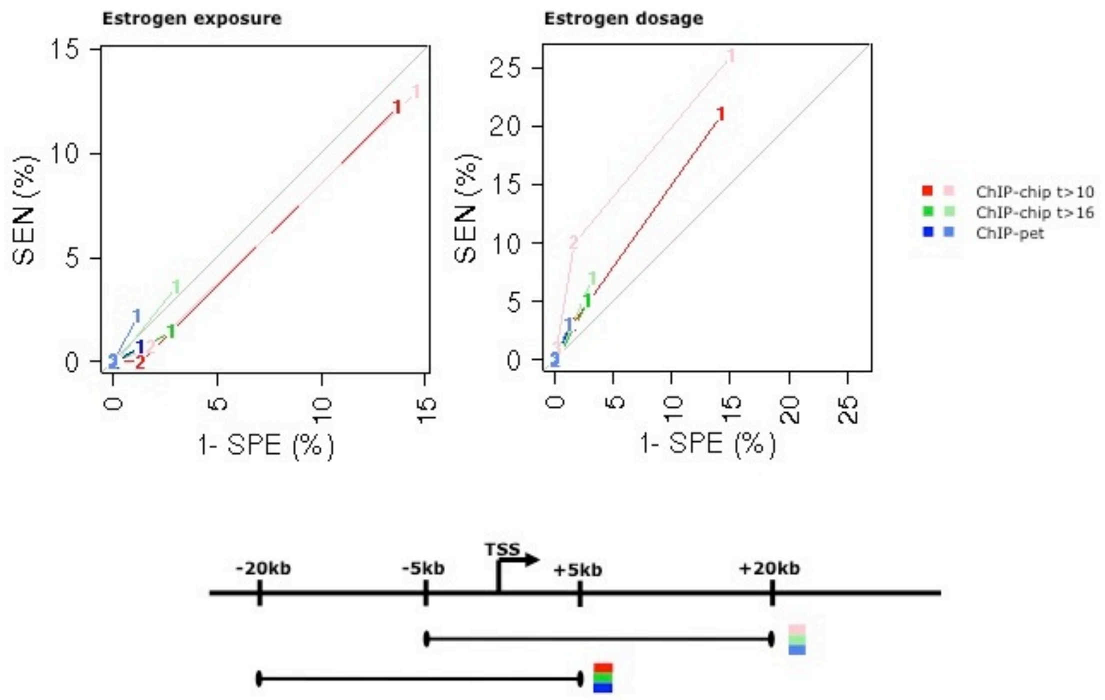

Fig. S3. ROC analysis for comparing the ability of upstream or downstream ChIP sites to predict induced genes.

Format is as in Fig. 3. In each experiment, the induced genes (positives) are taken as the 1% highest ranking transcripts. The remaining 99% are taken as the negatives. Shown are the estrogen exposure and estrogen dosage datasets (cf. Methods). It is noticeable that the estrogen exposure data shows random (points are on the diagonal) association, a finding that was confirmed by further analyses, e.g. ones as in Fig. 5.
