# Supplementary material for: Relationship between estrogen receptor α location and gene induction reveals the importance of downstream sites and cofactors
Source: BMC Genomics. 2009 Aug 18;10:381. doi: 10.1186/1471-2164-10-381 (PMC2907696; doi:10.1186/1471-2164-10-381)
Supplement: Additional file 4 — Supplemental Figure S4. ROC analysis to compare the ability of ChIP sites in variably sized windows to predict induced genes: cancer expression compendium and primary targets. [file 1471-2164-10-381-S4.pdf]

Supplemental Figure S4

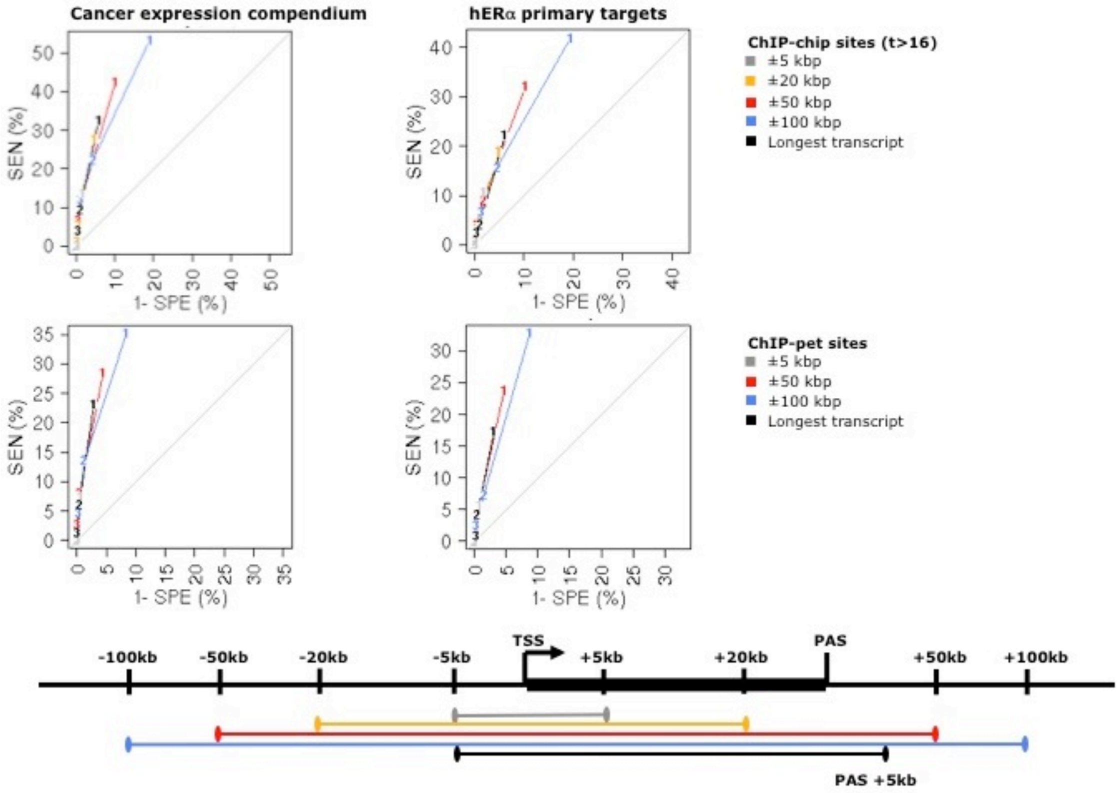

Fig. S4. ROC analysis to compare the ability of ChIP sites in variably sized windows to predict induced genes: cancer expression compendium and primary targets.

Positives and negatives are taken as in Fig. 3. Format is identical as for Fig. 4. but different sites are used: stringent ChIP-chip sites (t>16) in the upper row and ChIP-pet sites in the bottom row.
