# Supplementary material for: Relationship between estrogen receptor α location and gene induction reveals the importance of downstream sites and cofactors
Source: BMC Genomics. 2009 Aug 18;10:381. doi: 10.1186/1471-2164-10-381 (PMC2907696; doi:10.1186/1471-2164-10-381)
Supplement: Additional file 5 — Supplemental Figure S5. ROC analysis to compare the ability of ChIP sites in variably sized windows to predict induced genes: estrogen exposure and dosage datasets. [file 1471-2164-10-381-S5.pdf]

Supplemental Figure S5

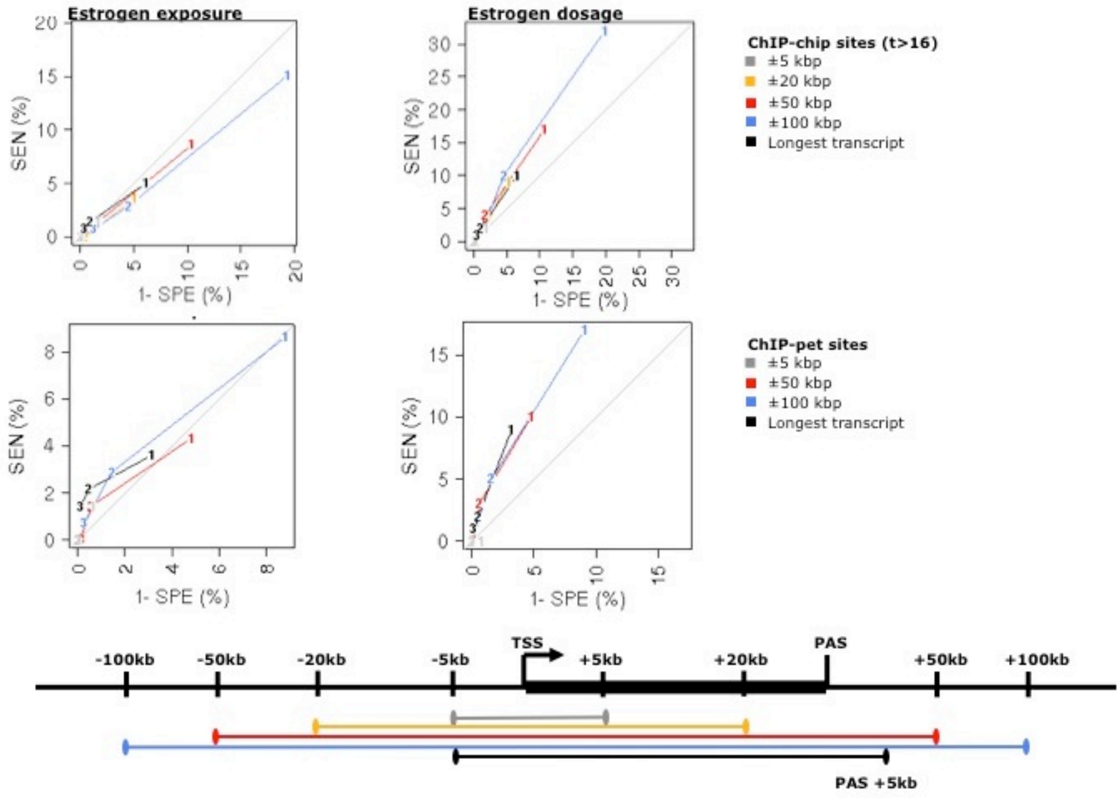

Fig. S5. ROC analysis to compare the ability of ChIP sites in variably sized windows to predict induced genes: estrogen exposure and dosage datasets.
Idem as Fig. S4 but with the estrogen exposure and estrogen dosage datasets (cf. Methods)
