# Supplementary material for: Relationship between estrogen receptor α location and gene induction reveals the importance of downstream sites and cofactors
Source: BMC Genomics. 2009 Aug 18;10:381. doi: 10.1186/1471-2164-10-381 (PMC2907696; doi:10.1186/1471-2164-10-381)
Supplement: Additional file 6 — Supplemental Figure S6. Comparison of log-likelihood distributions of the FOX factors PWMs for the ChIP sites with t>16. [file 1471-2164-10-381-S6.pdf]

Supplemental Figure S6

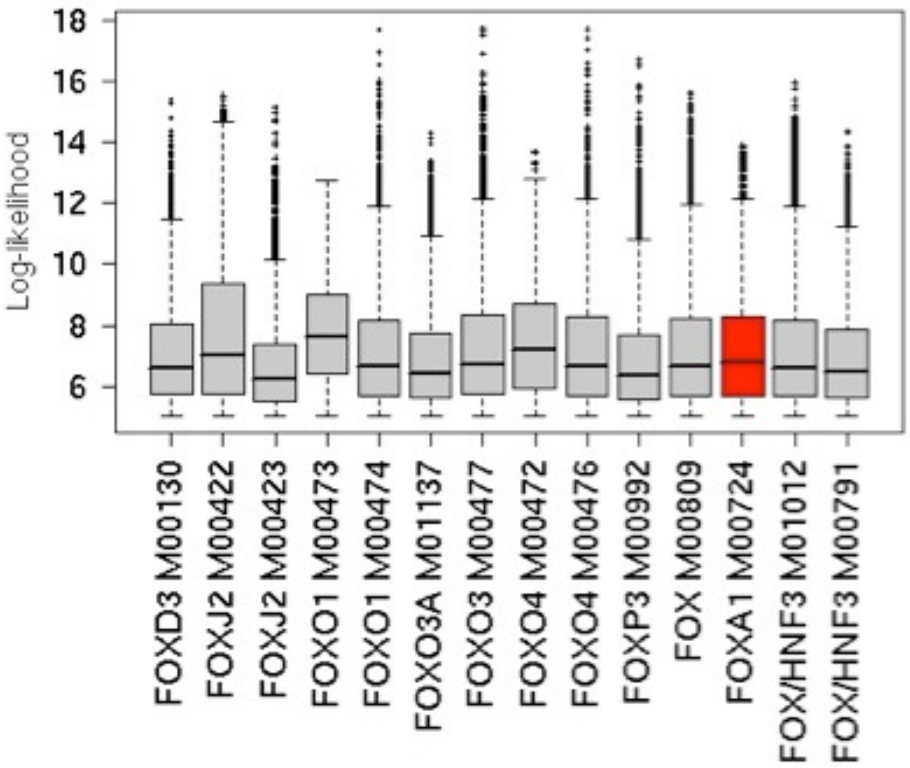

Fig. S6. Comparison of log-likelihood distributions of the FOX factors PWMs for the ChIP sites with t>16. The distribution of log-likelihoods of the FOXA1 PWM (in red) is no different from the distribution of other member of the FOX family. The PWMs have been taken from TRANSFAC.
