# Supplementary material for: Relationship between estrogen receptor α location and gene induction reveals the importance of downstream sites and cofactors
Source: BMC Genomics. 2009 Aug 18;10:381. doi: 10.1186/1471-2164-10-381 (PMC2907696; doi:10.1186/1471-2164-10-381)
Supplement: Additional file 7 — Supplemental Figure S7. Response to hERα increases in function of the number of ChIP sites along transcripts: only primary target dataset. [file 1471-2164-10-381-S7.pdf]

Supplemental Figure S7

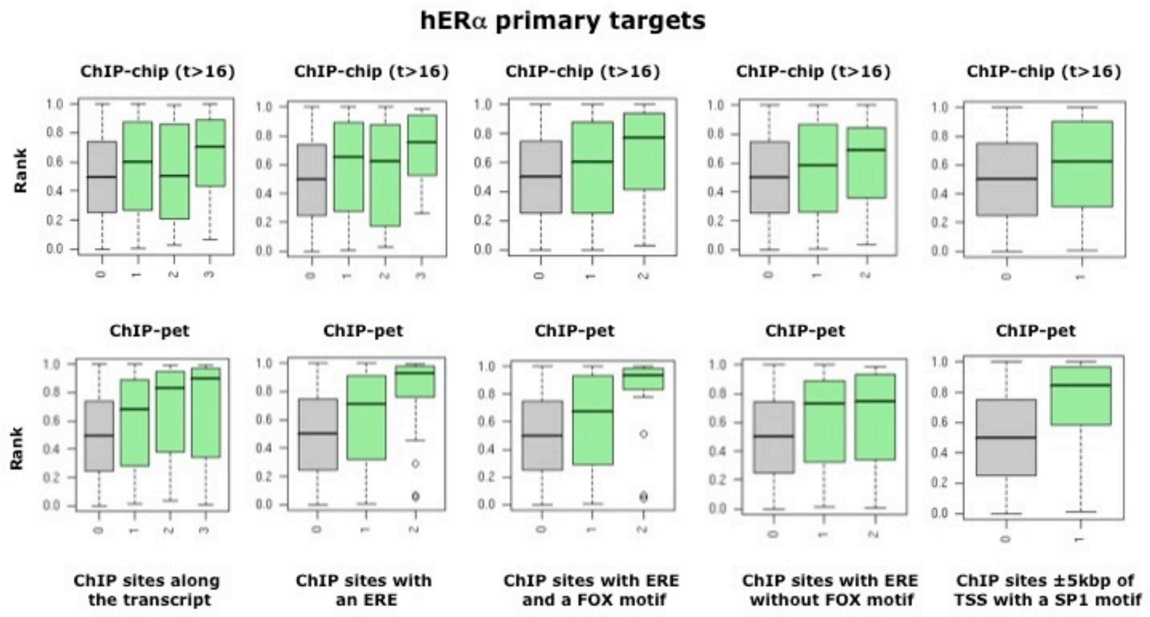

Fig. S7. Response to hERα increases in function of the number of ChIP sites along transcripts: only primary target dataset.
As in Fig. 5 and 6, we restrict the analysis to the hERα primary targets dataset.
Response to hERα increases in function of the number of ChIP sites along transcripts (first column). In the second, third and fourth columns, ChIP sites are further filtered according to the presence or absence of consensus elements for hERα (second column) or FOX (third and fourth column). In the fifth column the response to hERα is strengthened by the presence of a SP1 motif. A motif is assigned to a binding site if the occupancy, computed using posterior decoding, is greater than 0.5 (cf. Methods).
