# Supplementary material for: Relationship between estrogen receptor α location and gene induction reveals the importance of downstream sites and cofactors
Source: BMC Genomics. 2009 Aug 18;10:381. doi: 10.1186/1471-2164-10-381 (PMC2907696; doi:10.1186/1471-2164-10-381)
Supplement: Additional file 8 — Supplemental Figure S8. Sequence logos for hERα position weight matrices (PWMs). [file 1471-2164-10-381-S8.pdf]

Supplemental Figure S8

A

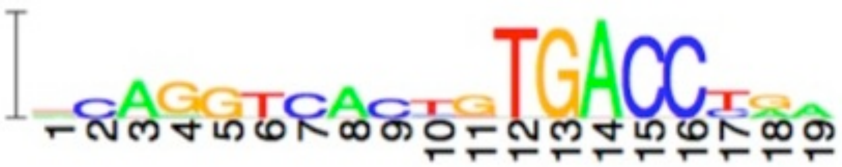

B

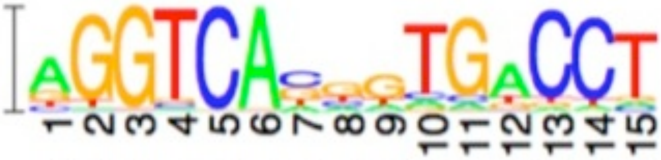

	1	2	3	4	5	6	7	8	9	10	11	12	13	14	15
A	56.0	1.9	0.2	2.1	0.2	98.5	13.4	30.9	14.6	8.3	7.1	55.5	2.3	3.1	3.3
C	3.6	0.4	0.2	2.9	99.0	0.2	39.9	15.9	26.5	8.6	7.1	27.6	90.6	86.9	16.9
G	36.5	88.9	98.7	2.9	0.6	1.0	38.2	39.0	46.8	3.6	84.6	7.1	3.3	0.6	3.8
T	3.8	8.8	1.0	92.1	0.2	0.4	8.4	14.2	12.1	79.5	1.2	9.8	3.8	9.4	76.0

Fig. S8. Sequence logos for hERα position weight matrices (PWMs).
The logos show the information content for each position (maximum is 2 bits).
A. TRANSFAC ER_Q6_V_M00191 PWM for hERα. The first half of the palindrome is weakly polarized making this logo unsuitable for high quality assessment of hERα binding.
B. Custom hERα PWM rederived from high confidence ChIP sites (t>16) by subselecting sequences showing the best matches to the TRANSFAC PWM.
